# Language phenotypes in children with sex chromosome trisomies

**DOI:** 10.12688/wellcomeopenres.14904.2

**Published:** 2019-01-28

**Authors:** Dorothy V. M. Bishop, Annie Brookman-Byrne, Nikki Gratton, Elaine Gray, Georgina Holt, Louise Morgan, Sarah Morris, Eleanor Paine, Holly Thornton, Paul A. Thompson

**Affiliations:** 1 Department of Experimental Psychology, University of Oxford, Oxford, Oxon, OX2 6GG, UK

**Keywords:** Trisomy X, Klinefelter syndrome, XYY syndrome, ascertainment bias, variability, psychometric testing, parent report

## Abstract

**Background** Sex chromosome trisomies (47,XXX, 47,XXY and 47,XYY) are known to be a risk factor for language disorder, but it is hard to predict outcomes, because many cases are identified only when problems are found.

**Methods** We recruited children aged 5-16 years with all three types of trisomy, and divided them into a High Bias group, identified in the course of investigations for neurodevelopmental problems, and a Low Bias group, identified via prenatal screening or other medical investigations. Children from a twin sample were used to compare pattern and severity of language problems: they were subdivided according to parental concerns about language/history of speech-language therapy into a No Concerns group (N = 118) and a Language Concerns group (N = 57). Children were assessed on a psychometric battery and a standardized parent checklist. After excluding children with intellectual disability, autism or hearing problems, the sample included 28 XXX, 18 XXY and 14 XYY Low Bias cases and 7 XXX, 13 XXY and 17 XYY High Bias cases.

**Results** Variation within each trisomy group was substantial: within the Low Bias group, overall language scores were depressed relative to normative data, but around one third had no evidence of problems. There was no effect of trisomy type, and the test profile was similar to the Language Concerns comparison group. The rate of problems was much greater in the High Bias children with trisomies.

**Conclusions** When advising parents after discovery of a trisomy, it is important to emphasise that, though there is an increased risk of language problems, there is a very wide range of outcomes. Severe language problems are more common in those identified via genetic testing for neurodevelopmental problems but these are not characteristic of children identified on prenatal screening.

Chromosome trisomies arise when an error of cell division leads to an egg or sperm that contains two copies rather than one copy of the chromosome. Trisomies that affect one of the autosomes are usually lethal, and in survivors they lead to marked physical and mental abnormalities. Trisomies of the sex chromosomes, however, have milder impacts, and often go undetected ( Printzlau *et al*., 2017). This complicates the study of the consequences of sex chromosome trisomies in two ways; first, it can be difficult to recruit large samples of cases, and second, those that are studied may not be representative of the population. In particular, there is a danger of overestimating the severity of impairment if we include cases where discovery of the trisomy was prompted by genetic testing to investigate developmental abnormalities.

In the 1960s, a multicentre project was initiated with the aim of evaluating the impact of sex chromosome trisomies in samples identified on newborn screening that were free from ascertainment bias ( Robinson *et al*., 1979). The three kinds of trisomy - trisomy X (47,XXX), Klinefelter’s syndrome (47,XXY) and 47,XYY karotypes were all found to be associated with neurodevelopmental problems, particularly affecting language and motor functions, see Leggett *et al*. (2010) for review. Nevertheless, in summarising implications for genetic counselling, Linden *et al*. (2002) concluded: ‘It is now known that these individuals are at an increased risk for developmental problems, but that most are in the normal range of development, and marked abnormality is not usually seen.’ (p 4)

To our knowledge, there have been no more newborn screening studies initiated in the past 50 years. One reason is practical: each of the three trisomy types has a prevalence ranging from 1 in 600 to 1 in 1000, so many thousands of cases need to be screened to identify even a small sample. Second, ethical issues are raised when a screening procedure identifies a trisomy in a newborn: while knowledge of the trisomy could be useful in helping parents take steps to ensure early intervention, it can also be damaging by creating anxiety ( Valentine, 1979).

It is now possible to detect sex chromosome trisomies in the foetus in the course of prenatal screening. Prenatal screening for Down syndrome was developed in the 1980s and led to cases of sex chromosome trisomy being detected as an incidental finding ( Cuckle & Maymon, 2016). Because prenatal screening is offered to older mothers, there is some bias in samples identified this way, but detection of the trisomy is not dependent on the child’s developmental outcome.

A study of neurodevelopmental of outcomes of prenatally-identified children was reported by Bishop *et al*. (2011). Outcome measures were based on standardized parental report, as it was deemed unethical to conduct direct testing of children who might not be aware of their trisomy. This study was consistent with the earlier newborn screening studies in finding a high rate of language difficulties in all three trisomies, but in addition, there was an elevated risk of autism spectrum disorder (ASD) diagnosis in boys with both XXY and XYY karyotypes. This had not been reported in the earlier prospective studies of newborns, possibly because diagnostic criteria for autism were far more stringent in the 20th century. A second group of children with trisomies detected postnatally was found to have a similar profile, but levels of all neurodevelopmental problems were higher, as would be expected in a group where there was ascertainment bias. In the course of this study, we established that 43% of children had been told about the trisomy. This suggested that a study that involved direct neurodevelopmental assessment of children would be feasible.

The association with language problems is intriguing because there are few genetic aetiologies that selectively impair language development. There are two reasons why it is of interest to compare the language phenotype of children with sex chromosome trisomies with that seen in children with developmental language disorder (DLD) of unknown origin. First, a comparative study could also help determine whether interventions that have been devised for children with DLD are also likely to be effective for children with a sex chromosome trisomy: ( Bishop *et al*., 2017). Second, if the similarities are not just superficial, then studying how an extra chromosome affects language development could help us understand aetiology of DLD ( Bishop & Scerif, 2011). Studies to date have suggested that there may be different profiles of impairment in the three types of trisomy, which could be related to the different genetic and hormonal impacts of an extra X or Y chromosome ( Bender *et al*., 1983), but we still have only a very limited body of data from samples free from ascertainment bias. It has also been suggested that an additional X chromosome could have more variable effects than an additional Y chromosome ( Skuse, 2018).

In 2011 we embarked on data collection for a new study of children with sex chromosome trisomies, including both pre- and post-natally identified cases. The goals were, first, to document the nature and range of language and behavioural difficulties in the three types of trisomy, and second, to test whether individual variation in outcomes could be predicted by variation in specific genetic variants. Here we report descriptive data relating to the first question, with a particular emphasis on language characteristics. Behavioural and psychiatric findings will be covered in a companion paper. To date we have not found any associations of phenotypes with variants in CNTNAP2 and NRXN1 genes; these analyses are reported elsewhere ( Newbury *et al*., 2018).

Our focus here is both on documenting whether there are reliable differences in language and cognitive outcomes for the three types of trisomy, and how far the pattern of language problems resembles that seen in children with developmental language disorder (DLD) who do not have any known genetic abnormalities. To minimize ascertainment bias, these analyses were restricted to children whose trisomy was either detected prenatally, or as an incidental finding during other medical investigations (referred to as a ‘Low Bias’ group). To address the latter question, we compared the results of trisomic children with those of children who had participated in a twin study of language development and disorders, and who had been given the same test battery. Finally, we considered how far trisomy outcomes are influenced by ascertainment bias, by comparing the Low Bias group with a High Bias group whose trisomies were discovered in the course of investigation of behavioural or neurodevelopmental problems.

The specific questions we considered were:

1. Does severity or variation in language profile differ across the three types of trisomy?

2. Is there a distinctive language and cognitive phenotype of children with sex chromosome trisomies, or do they resemble children with language problems who have the usual XY or XX karyotype?

3. How much does ascertainment bias affect the language and cognitive profiles of children with sex chromosome trisomies?

## Procedure

Ethical approval was obtained for the study in 2011 from the Berkshire NHS Research Ethics Committee (reference 11/SC/0096), and data collection started in August of that year, finishing in October 2016. Information sheets, consent forms and ethics approval documents are available on Open Science Framework.

## Participants

### Sex chromosome trisomy cases

We recruited 45 girls with XXX, 46 boys with XXY and 51 boys with XYY to the study, in the age range from 5 years 0 months to 15 years 11 months. 28% of the children had participated in the Bishop *et al*. (2011) study. Recruitment was via NHS Clinical Genetics centres, two support groups (Unique: the Rare Chromosome Support Group, and the Klinefelter Syndrome Association) and self-referral through social media. To be included in the study, the child had to be aware of their trisomy status and assent to take part after viewing an information video that explained what was involved in the study.

**Figure 1.**
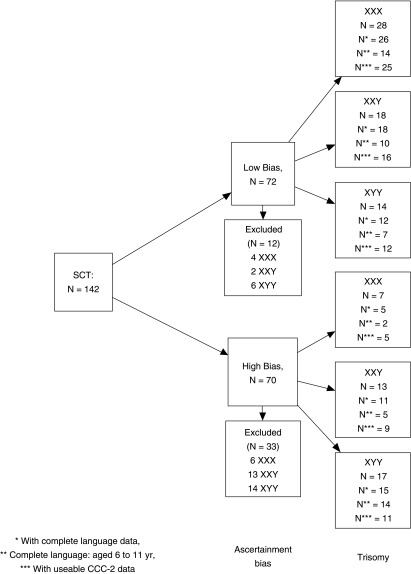
Numbers of children with sex chromosome trisomies included in different analyses.

During the initial telephone interviews, caregivers were asked how their child was diagnosed, in particular whether this followed postnatal testing motivated by neurodevelopmental/behavioural problems. The phenotype of such children may be more severe, potentially biasing the sample, and so, as shown in Figure 1, we grouped them prospectively as a High-risk-of-bias (High Bias) subgroup, comprised of 13 XXX, 26 XXY and 31 XYY cases. All other children formed the low-risk-of-bias (Low Bias) subgroup, with 32 XXX, 20 XXY and 20 XYY cases.

This latter group included 49 families who underwent amniocentesis: all but 4 parents provided a reason: 30 because of maternal age, 11 because nuchal screening or ultrasound scan indicated risk of a chromosome disorder, and 4 because of a family history of a chromosome disorder. A further 23 children had genetic testing for a range of medical conditions, including growth problems, failure to thrive, and failure to go through puberty (in the case of boys with XXY). Note that in referring to these as a ‘Low Bias’ group, we do not imply they are a representative sample of children with an extra sex chromosome: we make the more limited claim that the trisomy was not discovered as a result of the child manifesting language or behaviour difficulties. All genetic testing was done via the National Health Service, so the sample was not limited by parents’ ability to pay for a test.

There was an unanticipated imbalance between the three trisomy types in terms of the proportion of cases in the Low Bias group: 80% for XXX girls, 58% for XXY boys, and 45% for XYY boys; chi-square (2) = 8.73, p = 0.01.

### Exclusionary criteria

Given our current focus on language disorder, we excluded from the main analysis children who had a nonverbal ability scaled score (PIQ) more than 2 SD below the mean (2 Low Bias and 9 High Bias), those with a diagnosis of Autism Spectrum Disorder (ASD) (9 Low Bias and 23 High Bias), and those who failed a hearing screen (1 Low Bias and 1 High Bias). This enabled us to make a direct comparison of language scores with the twin sample, which also excluded children on this basis, and to see how far language problems were seen in children who did not have accompanying conditions that are likely to affect language function. However, note that this entails that our conclusions are based on a selected sample of trisomy cases. Where children who were excluded because of low IQ or ASD had completed the test battery, their individual data are shown in the plotted results, so the reader can form an impression of the impact of the exclusions on summary results. ASD in children with sex chromosome trisomies will be the focus of a companion paper.

### Missing data

Where a child had missing data on just one of the language/cognitive tests, that value was prorated from the mean of that child on the other measures. Likewise, when there was just one missing measure on reading-related tests, the value was prorated from other reading measures. 14 children had more missing data than this, and were excluded from the current analyses. This included 7 five-year-olds, for whom standard scores were not computable as they fell below the age range of norms. All but one case of missing data in children aged over 5 years came from the High Bias group, reflecting refusal or inability to attempt some tests. Norms for the CCC-2 extend across the full age range covered here, but 19 children had missing data because parents either did not complete the CCC-2 (N = 12), or because they failed the consistency check (N = 7), which is suggestive of invalid responses.

The numbers included in different analyses are shown in the flowchart in Figure 1. When comparing test profiles of trisomy cases with those of twins with language concerns (see below), we restricted consideration to children covering the same age range, i.e. 6 yr 0 months to 11 yr 11 months.

### Comparison group

Our second question involved comparing the language profile of children with trisomies with that of children with language disorders of unknown cause. The comparison sample consisted of twins recruited via fliers sent to primary schools around the UK, advertisements on our group’s website and via twins’ clubs. The age range for this sample was narrower than for the SCT cases. We aimed to recruit families with twin children aged between 6 years 0 months and 11 years 11 months, with over-representation of those where one or both twins had language or literacy problems. Further details are provided in Wilson and Bishop ( Wilson & Bishop, 2018)

Usually, language disorder would be diagnosed on the basis of language test scores, but if we took that approach, the result would be a foregone conclusion, in that we would use the same measures to define the independent variable (language status) and the dependent variables. To avoid this circularity, twin children were subdivided according to parental concern about language and history of speech and language therapy (SALT), rather than by language test scores, which were treated as dependent variables. Parental concern was coded from the initial interview, and used to divide the twin sample into a Language Concerns group with ongoing parental concerns about oral language (mild or severe) and the remainder (which included some cases where there had been transient concerns in preschool that had resolved, or where the concern affected only reading). The latter is referred to as the No Concerns group. In addition, children who had received speech and language therapy after the age of 4 years were included in the Language Concerns group.

To be included in the twin sample, neither twin could have a pre-existing diagnosis of autism spectrum disorder (ASD), or a serious long-term illness. In addition, children were excluded if an ASD diagnosis was confirmed after the study was completed (N = 1), if the child had a performance IQ score below 70 (N = 1) or failed a hearing screen (N = 4). We followed the CATALISE criteria for DLD ( Bishop *et al*., 2017), which meant that other diagnoses, such as dyslexia, attentional deficit hyperactivity disorder (ADHD), or dyspraxia, were not grounds for exclusion.

**Figure 2.**
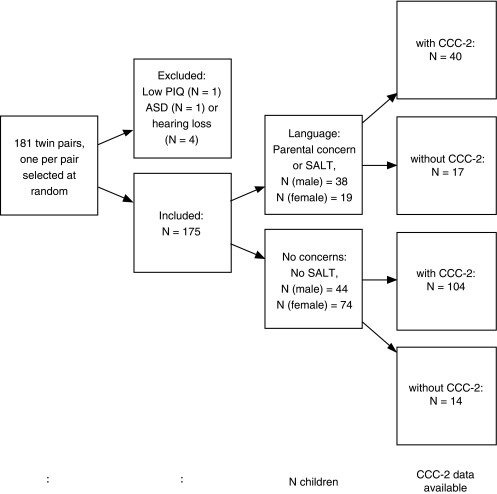
Numbers of twin children with and without language concerns.

One twin from each pair was selected at random to avoid dependencies in the data. The final sample consisted of 57 children in the language concerns group, and 118 children with no concerns. The latter group were used to derive a normative range against which to evaluate the other groups on language measures. Figure 2 shows the number of twin children included in the study, and the number for whom useable CCC-2 data were available.

## Methods

### Language, literacy and cognitive assessments

The assessment battery administered to the child is shown in Table 1.

**Table 1.** Psychometric assessment battery.

Instrument	Measure
Audiometry: screen at 25 dB (HL) for frequencies 500, 1000, 2000 and 4000 Hz.	Passed if average threshold in better ear was 30 dB or less
Wechsler Abbreviated Scale of Intelligence (WASI) ( Wechsler, 1999)	Matrices (nonverbal reasoning)
	Block Design (visuospatial skills)
	Vocabulary
Woodcock Johnson III Tests of Cognitive Abilities ( Woodcock *et al.*, 2007)	Verbal Comprehension
NEPSY: A Developmental Neuropsychological Assessment ( Korkman *et al.*, 1998)	Sentence Repetition
	Repetition of Nonsense Words
	Oromotor Sequences
Neale Analysis of Reading Ability – 2nd British edition (NARA-II) ( Neale, 1999)	Passage reading, giving measures of accuracy, comprehension and rate
Test of Word Reading Efficiency (TOWRE) ( Torgesen *et al.*, 1999)	Speeded reading of words (Sight Word Efficiency) and nonwords (Phonetic Decoding Efficiency)
Phonological Assessment Battery (PhAB) ( Frederickson *et al.*, 1997)	Picture Naming Test
	Digit Naming Test

Parents were asked to complete the Children’s Communication Checklist – 2 ( Bishop, 2003) and Social Responsiveness Scales ( Constantino, 2005) and return them by mail; the response rate was 86% for the trisomy families and 83% for the twin families. The SRS data and results from an online interview covering behavioural and psychiatric characteristics will be described in a companion paper.

Results from the assessments in Table 1 were converted to age-scaled scores on a common scale with mean 100 and SD 15. All tests had published norms covering the age range of the twin sample (6 to 11 years), but some did not extend outside this range. As explained in Newbury *et al*. (2018), where feasible, norms were extrapolated based on data from other samples encompassing the age range (see Appendix 2 on Open Science Framework project: https://osf.io/ae8yn/). For Oromotor Skills, however, extrapolated norms gave scaled scores that were well below the range of other tests. For the current analyses, therefore the No Concerns twin group was used to derive norms, using the regression of total correct on age to compute a standardized residual, which was then scaled to mean 100 and SD 15.

Our original intention was to use MANOVAs to test group differences that related to our research questions, but preliminary analysis showed that the MANOVA assumption of multivariate normality was not met when all 14 language/cognitive measures were entered into the analysis. Accordingly, we created four composites for the psychometric tests, by averaging scores as follows: Nonverbal ability (Matrices and Block Design), Core language (Vocabulary and Woodcock-Johnson Comprehension), Verbal production/memory (Sentence repetition, Nonword repetition, Oromotor sequences) and Literacy skills (all subtests from Neale Analysis of Reading Ability, Test of Word Reading Efficiency, and PhAB Rapid Naming). These composites mostly met criteria for multivariate normality within subgroups. Distributions of scores on the component tests for the different groups are shown in Supplementary Material, Figure S1. Multivariate normality was also an issue for the CCC-2 test data, so composites were formed based on three sets of scales, identified as Structural Language (scales A-C), Pragmatics (scales D-G), and Autistic Features (scales H-J). The means and SEs for the original 10 subscales are shown in Supplementary Material, Figure S2.

### Covariates

Two measures of socio-economic background that have been associated with language status were included in the analysis. These were:

a) Educational level of mother, transformed into an ordinal scale based on age at leaving full-time education/qualifications obtained, with points of 0 (prior to age 16 years), 1 (16 years/did GCSE or O-levels), 2 (18 years/ did A-levels), 3 (21 years, degree), 4 (postgraduate study).

b) An index of multiple deprivation based on postcode was obtained for those living in England from the website http://imd-by-postcode.opendatacommunities.org/. This uses local statistics from the Department for Communities and Local Government to rank 32,844 postcodes on the basis of a weighted sum based on income, employment, education, health, crime, housing and living environment. The rank score was converted to a z-score to give a normally-distributed variable, termed Neighbourhood Advantage index, by dividing by 32,844, before applying the qnorm function in R. A Neighbourhood Advantage index of zero (i.e. average) was assigned to 13 SCT cases and 4 twin pairs from Wales, Scotland and Northern Ireland, where postcode rankings were not available.

In addition, following a suggestion by Boada *et al*. (2009), we considered whether report of a positive family history of language problems was related to the language phenotype in the child. In an initial telephone interview with a parent, details of the mother, father and any siblings were recorded, and the informant was asked if each relative had any problems with hearing, speech, language or reading, and asked to elaborate if so. Family history was coded as positive if a relative was recorded as having definite evidence of language or literacy problems that had led to them requiring speech-language therapy or additional support at school. A three-point scale was used, with score of 0 for no family history, 1 for one affected relative, and 2 for two or more affected relatives.

### Procedure

Children were seen for an individual assessment at home or school. The language, reading and nonverbal ability tests ( Table 1) were given in an initial session lasting around 90 minutes, followed by an assessment of laterality, the results of which are described elsewhere ( Wilson & Bishop, 2018).

### Analysis

Table 2 shows the characteristics of the trisomy and twin groups on age, covariates and family history, for children included in the analysis of language test data.

**Table 2.** Characteristics of the trisomy and twin groups on background variables: age, mother’s educational level and Neighbourhood Advantage index.

Group	N	age (mo)	Mo. educ.	Neighbourhood Advantage	Family history
XXX: Low Bias	28	136 (46.7)	2 (0.96)	0.6 (0.82)	0.5 (0.7)
XXY: Low Bias	18	140 (43.4)	1.9 (1.06)	0.5 (0.86)	0.4 (0.61)
XYY: Low Bias	14	123 (42.4)	2.2 (0.89)	0.4 (0.77)	0.4 (0.51)
XXX: High Bias	7	117 (53.5)	1.4 (0.53)	-0.3 (1.14)	0.7 (0.49)
XXY: High Bias	13	132 (41.2)	1.8 (1.07)	0.6 (0.79)	0.6 (0.77)
XYY: High Bias	17	112 (33.5)	1.6 (1)	-0.2 (0.86)	0.6 (0.71)
Twin: No concerns	118	106 (19.3)	2.4 (0.93)	0.6 (0.95)	0.4 (0.64)
Twin: Language concerns	57	108 (18.9)	2.1 (0.9)	0.4 (1.17)	0.5 (0.68)

### Statistical comparisons

For each type of variable (Psychometric tests and parent report on CCC-2) three comparisons were conducted, corresponding to the three research questions, A) Comparison of the three trisomies for Low Bias group only; B) Comparison of all Low Bias trisomy cases aged 6-11 yr with the Language Concerns twin comparison group; C) Comparison of Low Bias and High Bias trisomy groups. A Bonferroni-corrected alpha level of .05/6 = .008 was adopted.

### Psychometric tests

**Figure 3.**
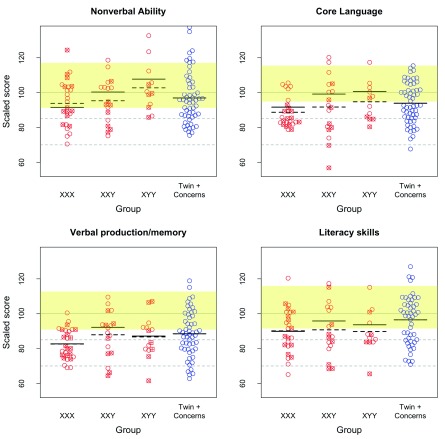
Distributions of scores on four clusters of psychometric tests for the Low Bias trisomy groups and the Language Concerns Comparison group. Open circles show cases in age range 6 to 11 yr. Solid line is mean for 6–11 yr olds, dashed line is mean for whole sample including those outside 6–11 yr age range. Yellow band is mean +/- 1 SD for No Concern comparison group.

**Figure 4.**
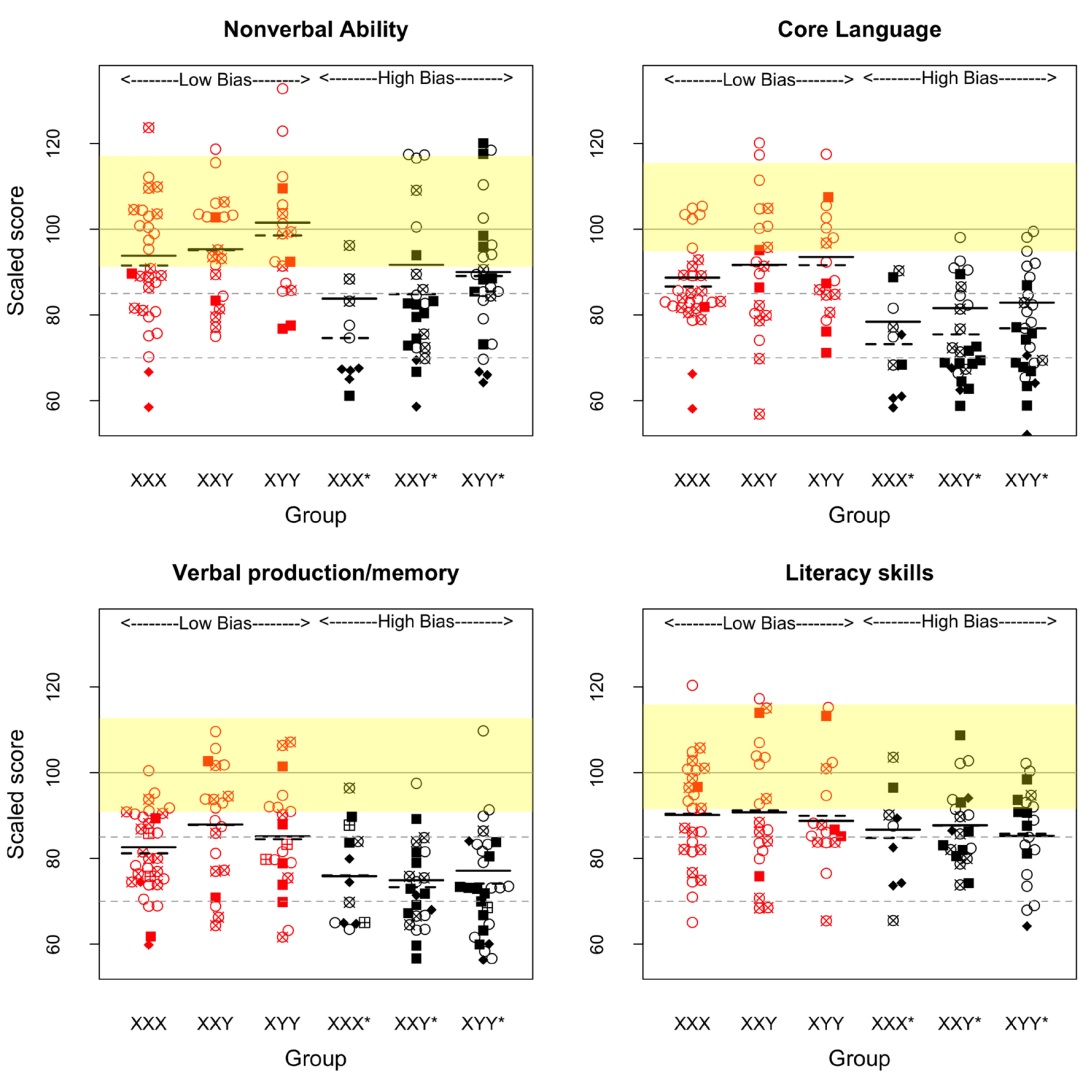
Distributions of scores on four clusters of psychometric tests for the Low Bias vs High Bias trisomy groups. Unfilled circles show cases in age range 6 to 11 yr, unfilled circles with a cross show older children, unfilled squares with cross show 5-year-olds. Cases with ASD (filled squares) and intellectual disability (filled diamonds) are included in the plot, but were excluded from the analysis comparing bias groups. Asterisk denotes High Bias group. Solid lines are group means for children included in MANOVA; dashed lines show means with ASD/ID cases included. Yellow band is mean +/- 1 SD for No Concern comparison group.

***A. Comparison of three trisomy groups (Low Bias group only). ***As a first step, data from the psychometric composites were plotted to see the range and distribution of scores for the three Low Bias trisomy groups, as shown in the red beeswarm plots in Figure 3. (These plots do not show scores for children who were excluded because of intellectual disability or ASD, but note that scores for those children are shown below in Figure 4). The dotted horizontal bars show the mean for each group. The yellow shaded area shows the range covered by mean +/- 1 SD for the No Concerns twins. The distribution of blue points, corresponding to the Language Concerns twins will be discussed below (analysis B).

An initial impression from these plots is that there is variation from test to test in severity of impairment, but on all measures, the most striking feature is a wide spread of scores, with some children scoring above average and others well below. Although the mean test scores vary from trisomy to trisomy, the within-group variation is much greater than between group variation. This pattern is also evident in data from the individual tests making up the composites (Supplementary Material, Figure S1).

MANOVA was used to test whether there were reliable differences between the three trisomy groups on the first three composite measures, after taking covariates (Mother’s educational level and Neighbourhood Advantage index) into account. These tests are based on the means shown in dotted lines, including all ages. No effect of trisomy type was found (see Table 3, row A). In addition, to test the prediction by Skuse (2018) of smaller variance in the XYY group compared with the groups with an extra X chromosome, F-tests were conducted, but on no measure was there any support (all p-values above .05).

**Table 3.** Results 3 MANOVAs (A, B and C) testing for overall group differences on three composites from psychometric tests (Nonverbal Ability, Core Language and Verbal Production/Memory), with covariates of mother’s education and Neighbourhood Advantage index.

Comparison	Effect	Wilks λ	F	df1	df2	p
A. Low Bias: XXX (26) vs XXY (18) vs XYY (12)	Group	0.885	1.030	6	98	0.409
	Mo. education	0.912	1.580	3	49	0.207
	Neighbourhood Advantage	0.890	2.010	3	49	0.124
B. Trisomies (31) vs. Lang concern twins (55): aged 6–11	Group	0.967	0.900	3	79	0.446
	Mo. education	0.802	6.490	3	79	0.001
	Neighbourhood Advantage	0.990	0.270	3	79	0.847
C. Low Bias (56) vs. High Bias (31)	Group	0.814	6.170	3	81	0.001
	Mo. education	0.940	1.710	3	81	0.171
	Neighbourhood Advantage	0.953	1.330	3	81	0.270

The literacy skills score had more missing data than other scores, and so was analysed separately using ANOVA, again revealing no effect of trisomy type, F (1 , 53) = 0.001 , p = 0.97.

***B. Comparison of combined trisomy cases aged 6–11 yr (Low Bias) with Language Concern twin comparison group.*** For this analysis, we combined all trisomy Low Bias cases aged 6–11 years (open circles in Figure 3) to compare scores on the first three psychometric composites with the twin comparison group with language concerns. Table 3, row B, shows the MANOVA result for this comparison, which concerns the means shown as solid lines. Again there was no overall effect of group. The only factor affecting scores was mother’s education, with each additional point on the scale associated with an increase of 4.40 points in average score on psychometric tests.

An ANOVA on the Literacy composite revealed that the Trisomy and Language Concerns groups were comparable in levels of impairment, F (3 , 74) = 0.74 , p = 0.53.

***C. Comparison of effect of bias group: trisomy cases only.*** Figure 4 shows the distributions of scores on the four composites for children in the two ascertainment risk groups. For this analysis, we considered the effect of bias group, combining across trisomy type, again entering the first three composites into a MANOVA. As shown in Table 3, row C, there was a strong effect of ascertainment bias, with the High Bias group having lower test scores. It is evident from inspection of Figure 4 that this effect would have been larger still if we had included the cases with ASD and intellectual disability, who were more common in the High Bias group and tended to have low test scores.

The same dataset was used to explore the relationship with family history of language problems, by running linear regressions for each of the four composite measures, with the family history score as a predictor. In no case did the association prove statistically robust (all p-values > .05, maximum proportion variance explained = 0.03).

### Children's Communication Checklist - 2

Completion rates for the CCC-2 showed some variation by group: the checklist was completed by parents of 94.40% of the Low Bias and 77.80% of the High Bias trisomy group, and by 81.90% of the parents of No Concern twins and 76.40% of parents of Language Concern twins. Data was not useable for 5.10% of checklists which failed the consistency check: this is a criterion based on comparing scores for items describing strength and difficulties: if raw means are similar for both, this suggests that the respondent has not appreciated the need to change the polarity of responses between the two sets.

To establish potential bias introduced by those with missing or unusable data, we conducted a logistic regression analysis, with CCC-2 data coded as 1 (useable data) vs 0 (missing or inconsistent), and with maternal educational level, single parent status, trisomy status, and a composite measure of the child’s language status (language factor from Newbury *et al*., 2018) as predictors. Availability of CCC-2 data not predicted by parental characteristics (mother’s education or single parent status), but was predicted by whether the child came from the twin or trisomy group, and by severity of language problems. The lower response rate by parents of twins might be explained by the fact that checklist completion was more onerous for them, as they were asked to complete a checklist for each twin. As shown in Supplementary Table S1 and Supplementary Figure S3, the impact of child’s language status reflected that CCC-2 was less likely to be completed when the child had more severe language difficulties. Thus the CCC-2 results may underestimate the extent of communication difficulties in both samples.

**Figure 5.**
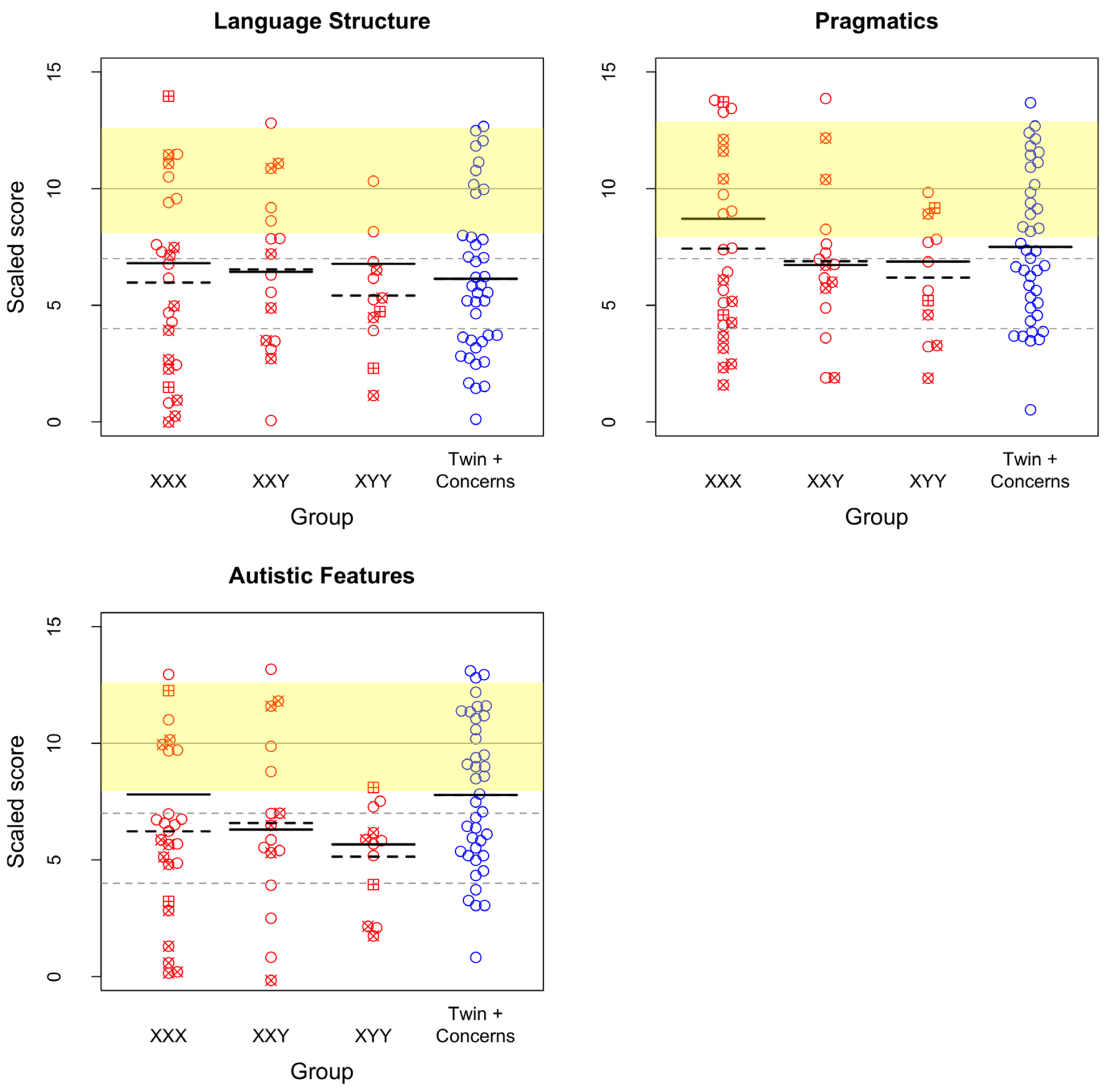
Mean scores on the three CCC-2 composites for trisomy and comparison groups. The yellow shaded band shows mean +/-1 SD for the No Concerns twin group. For trisomies, dashed lines show mean for whole group, and solid lines show means for children aged 6 to 11 years.

Mean scores on the three CCC-2 composites are shown for the Low Bias and Language Concerns groups in Figure 5. As expected, the No Concerns comparison group (range +/- 1 SD shown as yellow shading) has mean scores close to the normative mean of 10.

**Figure 6.**
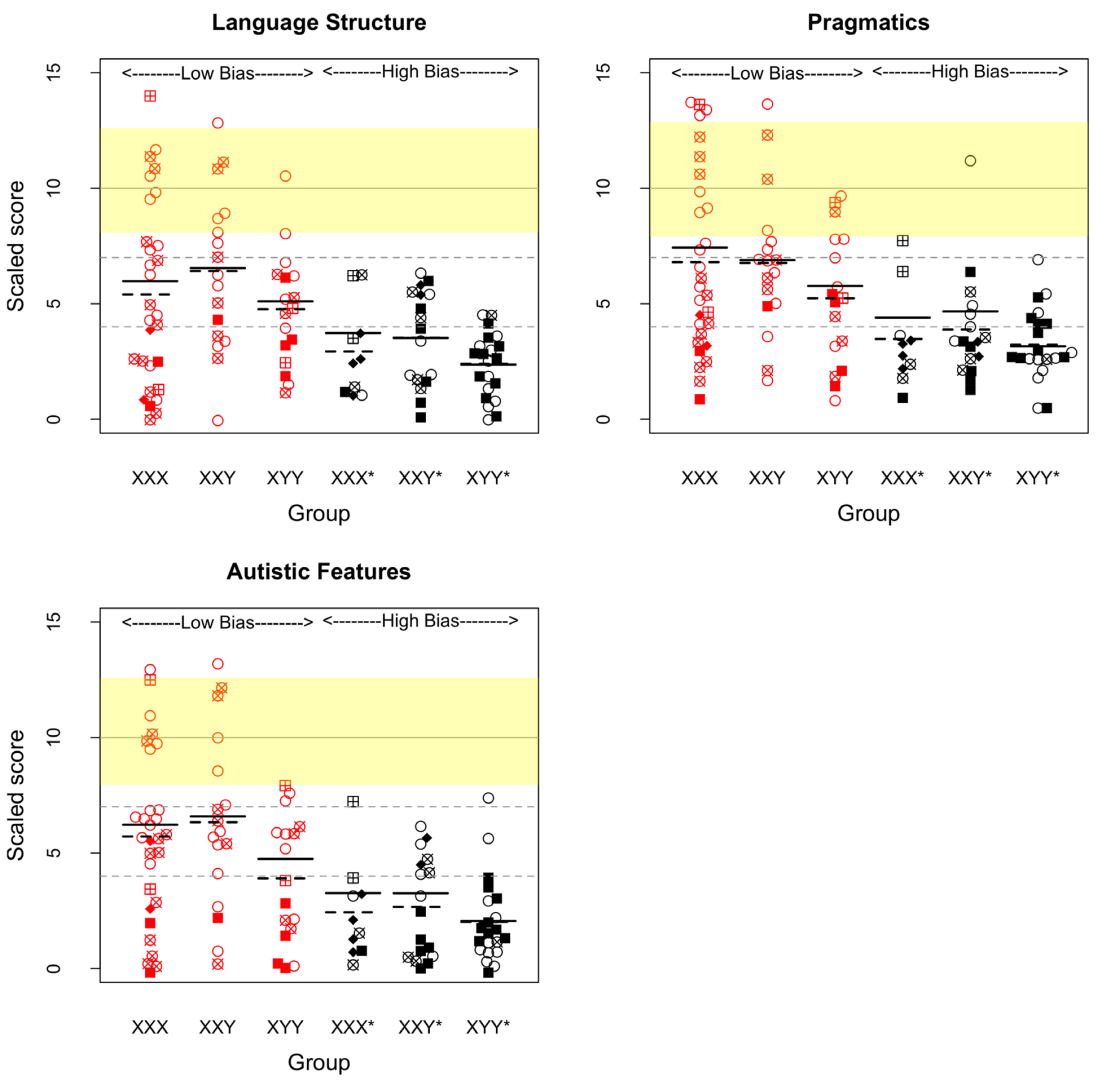
Mean scores on the three CCC-2 composites for Low Bias vs High Bias trisomy groups, with the latter denoted by asterisks. For trisomies, solid lines show mean for children included in MANOVA, and dashed lines show means for with cases of ASD or low IQ included. The yellow shaded band shows mean +/-1 SD for the No Concerns twin group.

Figure 6 contrasts CCC-2 composites for the Low Bias and High Bias trisomy groups. Here, the means are shown with and without cases of ASD and low IQ included: the MANOVA excluded these cases, focusing on differences in the means shown as solid lines. As with the psychometric tests, the scores were substantially lower for the High Bias groups, although there was a wide range in all three trisomies.

MANOVAs were conducted in the same way as for the psychometric test composites, again showing no effect of trisomy type within the Low Bias sample ( Table 4, row A), no difference between the combined trisomy group and the Language Concern twin comparison group (row B), but a substantial impairment in High Bias trisomy cases relative to the Low Bias cases (row C). For the Low Bias groups, the plot suggested there might be lower variance for the XYY children, in line with prediction by Skuse (2018), but p-values for F-tests comparing the variance of the XYY cases with the combined XXX and XXY cases were greater than .05 for all composites.

**Table 4.** Results for three MANOVAs comparing groups on the CCC-2 composite scores.

Comparison	Effect	Wilks λ	F	df1	df2	p
A. XXX (29), XXY (17), XYY (17)	Group	0.862	1.260	6	98	0.282
	Mo. education	0.914	1.530	3	49	0.219
	Neighbourhood Advantage	0.963	0.630	3	49	0.602
B. All SCT vs Lang concern twins	Group	0.869	3.210	3	64	0.029
	Mo. education	0.867	3.260	3	64	0.027
	Neighbourhood Advantage	0.928	1.660	3	64	0.185
C. Bias	Bias	0.751	10.370	3	94	0.000
	Mo. education	0.980	0.650	3	94	0.583
	Neighbourhood Advantage	0.918	2.800	3	94	0.044

### Number of language tests below cutoff

In a final analysis, we focused on rates of impairment rather than mean scores. There have been various attempts to operationalise diagnostic criteria for language disorder, e.g.( Tomblin *et al*., 1996). Although an overall language test composite can be used for this purpose, this may miss cases who have an uneven profile, with striking deficits in just a few aspects of language function. To get a better impression of the nature of language deficits in the trisomy and comparison groups, we selected five language measures: Vocabulary, Comprehension, Sentence Repetition, Nonword Repetition, and Oromotor Sequences, and categorised each case according to whether their score was more than 1 SD below the population mean (i.e. 85 or less on the rescaled scores). This analysis did not include the cases meeting exclusionary criteria. The percentages of children in each group who scored this low on between 0 and 5 tests is shown in Table 5.

**Table 5.** Percentages of children meeting criteria for impairment (score < 86) on a given number of tests (T0 to T5).

Group	N	T0	T1	T2	T3	T4	T5
Comparison							
No concerns	118	60.2	26.3	7.6	5.9	0	0
Language concerns	57	22.8	24.6	14	19.3	8.8	10.5
Trisomy: Low Bias							
XXX	28	10.7	14.3	14.3	28.6	28.6	3.6
XXY	18	27.8	16.7	16.7	22.2	0	16.7
XYY	14	0	42.9	21.4	21.4	14.3	0
Trisomy: High Bias							
XXX	7	0	14.3	14.3	14.3	14.3	42.9
XXY	13	0	7.7	7.7	15.4	38.5	30.8
XYY	17	0	17.6	11.8	11.8	35.3	23.5

Another indicator of language impairment is having a General Communication Composite (GCC) on the CCC-2 of 55 or less. The percentages of twins rated this low was 5.1% for the No Concerns group and 40.4% for the Language Concerns group. Among children with sex chromosome trisomies, 56.7% of the Low Bias group and 73.0% of the High Bias group were rated this poorly.

## Discussion

This study confirmed that there is a high rate of language problems among children with sex chromosome trisomies of all three kinds. In this relatively small sample, there were no consistent differences in the severity or profile of language problems between those with XXX, XXY and XYY karyotypes. In their study of neonatally identified cases, Bender *et al*. (2001) noted that females with XXX (N = 10) were more impaired than males with XXY (N = 11), but the conclusion was not based on direct comparison of the two groups and the sample size was small. An unanticipated finding from our study was that girls with XXX were more likely than the XXY and XYY boys to come from the Low Bias group, suggesting that fewer of them had problems that brought them to clinical attention. Ross *et al*. (2009) found that boys with XYY had more pervasive and severe language problems than boys with XXY karyotype, but they noted that the former group included more postnatally identified cases, so ascertainment bias could affect findings. Although it is possible that karyotype differences would emerge with a larger sample, a striking feature of our data, and those of Bender *et al*. (2001), is the wide range of variation within each type of trisomy, which swamps any trisomy-specific effect. Indeed, for children selected in a manner that reduced ascertainment bias, we found that around one third of cases scored in the same range as a comparison group without problems, in terms of having at most one low score on a set of five language tests. A similar proportion scored within the normal range on the overall index from parental report, i.e. the General Communication Composite of the Children’s Communication Checklist-2. Furthermore, for ethical reasons, we were only able to study children who knew about their sex chromosome trisomy: we know from our previous study that these tend to be children with more severe problems, where the trisomy may be disclosed in order to help the child understand about their difficulties ( Gratton *et al*., 2016). It is therefore likely that even our Low Bias group may overestimate the extent of language difficulties in childhood, because those without any difficulties would be less like to take part. On the other hand, the data reported here excluded 12 children from the Low Bias sample because of intellectual disability, ASD, or hearing problems, and so it needs to be borne in mind that had these cases been included, an even wider range of scores would have been observed. Skuse (2018) suggested that we might expect to see more variable expression of genes from an additional X chromosome than from the Y chromosome, in which case the language phenotype might have a narrower range in the XYY group ; this prediction was not supported. Note, however, that detection of reliable differences in variance between groups would require substantial sample sizes. Even for detection of mean group differences, our sample size was too small to detect any but large effects of karyotype: for comparisons of XXX+XXY vs XYY we had 80% power to detect an effect size on psychometric composites of .83, and on CCC-2 an effect size of .97. We cannot therefore conclude from the current study that there are no sex-chromosome-specific influences, only that if they do exist, they are not large, and are superimposed on substantial variation from other sources. Because of the difficulty of recruiting large samples, and the difficulty of controlling sampling biases, we will need to combine samples across different research groups to get a clear answer to the question of whether an additional X or Y has consistently different effects.

The twin sample was subdivided to provide a way of assessing how far the language problems in those with sex chromosome trisomies resembled those of children who had language problems in the absence of any known neurobiological condition. Allocation to the language concerns group was made purely on the basis of parental report: either there was ongoing concern about the child’s language skills and/or that the child had speech and language therapy after the age of 4 years. As can be seen in Figure 3 and Figure 5, although the overall means were below average, many children selected this way did not have obvious language problems on the test battery used here. This mismatch between parental report and test performance is reminiscent of findings by Broomfield & Dodd (2004) who found that around 10% of children on the caseload of a speech-language therapy service had normal range performance on language assessment. This suggests that parents may be concerned about relatively minor problems that are not clinically important, or that are transient and have resolved by the time we assessed the child. It could also be that the battery used here was not sensitive to the kinds of difficulties that children experienced. For instance, some children receive speech and language therapy for problems with articulation, voice or fluency, and those problems would not necessarily be detected on our assessment battery. In the sample of Broomfield & Dodd (2004) speech difficulties were the most common type of problem.

Insofar as it was possible to compare the Language Concerns group with the trisomy cases, the profile and severity of their problems appeared similar. Thus, we did not detect any distinct phenotypic signature of a sex chromosome trisomy.

When we turn to consider children whose trisomy was discovered in the course of investigation for neurocognitive or behavioural difficulties (i.e. the High Bias group), we find a substantially higher rate of language problems. Furthermore, 33 children were excluded from the High Bias sample because of intellectual disability, ASD or hearing problems, and a further 3 of the remaining 37 children from this group were excluded from analysis because of missing language test data due to refusal or inability. Thus the language test scores shown here apply only to those who could complete the test battery and did not have additional problems. These results agree with those of Wigby *et al*. (2016), who found substantial differences in neurodevelopmental outcomes for girls with trisomy X, depending on whether the trisomy was identified prenatally or postnatally. It is not surprising to find that children whose trisomy was identified in the course of investigations for developmental disorders should have high rates of problems, but this result reinforces the message that, when advising parents of likely outcomes of children in whom a sex chromosome trisomy is adventitiously discovered, it is important to focus on results from samples that minimise this kind of ascertainment bias ( Linden *et al*., 2002).

There are several possible factors that might account for the range of variation in phenotypes seen in sex chromosome trisomies. One possibility is that the impact of a sex chromosome trisomy might be influenced by environmental background. This idea has been raised by Bender *et al*. (1987). We included measures of environmental background - maternal years of education and neighbourhood advantage - in our analyses, and found evidence of an impact of maternal education on psychometric scores in one analysis (see Table 4, Analysis B), though this effect was not evident in an analysis that included only children with a sex chromosome trisomy. In a review of Klinefelter syndrome, Boada *et al*. (2009) proposed that family history of language problems might affect severity of language phenotypes, but we found no evidence for this in the current sample.

Elsewhere we have proposed that genetic variants on autosomes may interact with neurodevelopmental consequences of extra gene product from sex chromosomes, so there is amplification of impact from variants that usually have only a mild effect. To date we have not been able to identify such effects ( Newbury *et al*., 2018). This does not, of course, rule out the possibility that a mechanism of this kind does operate, but involving different genes. In future work, we plan to broaden the scope of our search for such genetic mechanisms. It is worth noting, however, the suggestion by Beach *et al*. (2017), that aneuploidy itself may be a cause of phenotypic heterogeneity. Most of their evidence comes from studies in yeast, where they found high variability in cell cycle progression among cells with identical aneuploidies, as well as variable response to environmental stress. They argued this tendency for gain or loss of a chromosome to cause genetic instability and increased variability in the phenotype may extend to mammals. They found wide variation in inbred mice with genetically engineered trisomy 19, despite a uniform genetic background and environment. If this applies to human sex chromosome trisomies, then a search for genetic or environmental correlates of phenotypic variation could prove fruitless.

Where a sex chromosome trisomy is discovered on prenatal screening, parents will be anxious to know the implications for the child’s development. The results reported here illustrate the very wide range of outcomes that can be seen in children with an extra X or Y chromosome. Some children in our sample had no measurable language difficulties, while others were severely impaired. In interpreting these findings, it is also important to bear in mind that, even if we consider just the Low Bias group, we may over-estimate the rate of impairment, if parents are more motivated to take part if their child has problems, and/or if the child’s eligibility for the study (in terms of knowing about the trisomy) is affected by level of impairment. These considerations make advising parents challenging, as prediction of individual outcomes is impossible: the most that can be said in our current state of knowledge is that there is a clear risk that the child will have language problems that may interfere with daily life, social interaction and progress in school, but that such problems are by no means inevitable. Most children attended mainstream school and it was not uncommon to find language skills in the normal range. Where children had language deficits not accompanied by ASD or intellectual disability, they tended to be of a range and severity similar to those seen in other children who have developmental language disorder (i.e. in the absence of any known biological aetiology). This reinforces the advice given by Linden *et al*. (2002) that children with sex chromosome trisomies do not need special interventions, as their difficulties with speech, language and learning are similar to those of other children with typical chromosomes.

## Data availability

Data and analysis scripts are available on Open Science Framework: https://osf.io/u2c7d/, DOI: https://doi.org/10.17605/OSF.IO/U2C7D ( Bishop, 2018). Data are available under the terms of the Creative Commons Zero "No rights reserved" data waiver (CC0 1.0 Public domain dedication).

## Supplementary material

- Distributions of scores on individual psychometric tests (Figure S1)
- Means for CCC-2 scales (Figure S2)
- Prediction of CCC-2 checklist completion from child and parent variables (Figure S3 and Table S1)

## References

[ref-1] Beach RR Ricci-Tam C Brennan CM: Aneuploidy causes non-genetic individuality. *Cell.* 2017;169(2):229–242.e21. 10.1016/j.cell.2017.03.021 28388408 PMC5441241

[ref-2] Bender B Fry E Pennington B: Speech and language development in 41 children with sex chromosome anomalies. *Pediatrics.* 1983;71(2):262–267. 6823432

[ref-3] Bender BG Linden MG Harmon RJ: Neuropsychological and functional cognitive skills of 35 unselected adults with sex chromosome abnormalities. *Am J Med Genet.* 2001;102(4):309–313. 10.1002/ajmg.1490 11503155

[ref-4] Bender BG Linden MG Robinson A: Environment and developmental risk in children with sex chromosome abnormalities. *J Am Acad Child Adolesc Psychiatry.* 1987;26(4):499–503. 10.1097/00004583-198707000-00006 3654500

[ref-51] Bishop DVM: Language and behavioural phenotypes in children with sex chromosome trisomies.2018 10.17605/OSF.IO/U2C7D PMC6376256 30815537

[ref-5] Bishop DVM: The Children's Communication Checklist, version 2 (CCC-2). London: Pearson.2003.

[ref-6] Bishop DV Jacobs PA Lachlan K: Autism, language and communication in children with sex chromosome trisomies. *Arch Dis Child.* 2011;96(10):954–959. 10.1136/adc.2009.179747 20656736 PMC3182523

[ref-7] Bishop DV Scerif G: Klinefelter syndrome as a window on the aetiology of language and communication impairments in children: the neuroligin-neurexin hypothesis. *Acta Paediatr.* 2011;100(6):903–907. 10.1111/j.1651-2227.2011.02150.x 21418292 PMC3107947

[ref-8] Bishop DVM Snowling MJ Thompson PA: Phase 2 of CATALISE: a multinational and multidisciplinary Delphi consensus study of problems with language development: Terminology. *J Child Psychol Psychiatry.* 2017;58(10):1068–1080. 10.1111/jcpp.12721 28369935 PMC5638113

[ref-10] Boada R Janusz J Hutaff-Lee C: The cognitive phenotype in Klinefelter syndrome: a review of the literature including genetic and hormonal factors. *Dev Disabil Res Rev.* 2009;15(4):284–294. 10.1002/ddrr.83 20014369 PMC3056507

[ref-11] Broomfield J Dodd B: Children with speech and language disability: caseload characteristics. *Int J Lang Commun Disord.* 2004;39(3):303–324. 10.1080/13682820310001625589 15204443

[ref-12] Constantino JN: The Social Responsiveness Scale. Los Angeles: Western Psychological Services.2005.

[ref-13] Cuckle H Maymon R: Development of prenatal screening--A historical overview. *Semin Perinatol.* 2016;40(1):12–22. 10.1053/j.semperi.2015.11.003 26764253

[ref-15] Frederickson N Frith U Reason R: Phonological assessment battery (PhAB). Windsor: NFER-Nelson.1997 Reference Source

[ref-40] Gratton NC Myring J Middlemiss P: Children with sex chromosome trisomies: parental disclosure of genetic status. *Eur J Hum Genet.* 2016;24(5):638–44. 10.1038/ejhg.2015.168 26306644 PMC4930078

[ref-16] Korkman M Kirk U Kemp SI: NEPSY: A developmental neuropsychological assessment. San Antonio: Psychological Corporation.1998 Reference Source

[ref-17] Leggett V Jacobs P Nation K: Neurocognitive outcomes of individuals with a sex chromosome trisomy: XXX, XYY, or XXY: a systematic review. *Dev Med Child Neurol.* 2010;52(2):119–129. 10.1111/j.1469-8749.2009.03545.x 20059514 PMC2820350

[ref-32] Linden MG Bender BG Robinson A: Genetic counseling for sex chromosome abnormalities. *Am J Med Genet.* 2002;110(1):3–10. 10.1002/ajmg.10391 12116264

[ref-18] Neale MD: Neale Analysis of Reading Ability Second Revised British Edition. London: GL Assessment.1999.

[ref-19] Newbury DF Simpson NH Thompson PA: Stage 2 Registered Report: Variation in neurodevelopmental outcomes in children with sex chromosome trisomies: testing the double hit hypothesis [version 1; referees: 2 approved]. *Wellcome Open Res.* 2018;3:85. 10.12688/wellcomeopenres.14677.1 30271887 PMC6134338

[ref-20] Printzlau F Wolstencroft J Skuse DH: Cognitive, behavioral, and neural consequences of sex chromosome aneuploidy. *J Neurosci Res.* 2017;95(1–2):311–319. 10.1002/jnr.23951 27870409

[ref-22] Robinson A Puck M Pennington B: Abnormalities of the sex chromosomes: a prospective study on randomly identified newborns. In A. Robinson, HA. Lubs, & D. Bergsma (Eds.) *Sex Chromosome Aneuploidy: prospective studies on children*. *Birth Defects Orig Artic Ser*.1979;15(1):203–241. 444642

[ref-23] Ross JL Zeger MP Kushner H: An extra X or Y chromosome: contrasting the cognitive and motor phenotypes in childhood in boys with 47,XYY syndrome or 47, XXY Klinefelter syndrome. *Dev Disabil Res Rev.* 2009;15(4):309–317. 10.1002/ddrr.85 20014371 PMC2876236

[ref-24] Skuse D: Referee Report For: Stage 1 Registered Report: Variation in neurodevelopmental outcomes in children with sex chromosome trisomies: protocol for a test of the double hit hypothesis [version 1; referees: 1 approved, 2 approved with reservations]. *Wellcome Open Res.* 2018;3:10 10.21956/wellcomeopenres.15031.r31601 29744390 PMC5904730

[ref-25] Tomblin JB Records NL Zhang X: A system for the diagnosis of specific language impairment in kindergarten children. *J Speech Hear Res.* 1996;39(6):1284–1294. 10.1044/jshr.3906.1284 8959613

[ref-26] Torgesen JK Wagner R Rashotte C: Test of Word Reading Efficiency (TOWRE). New York: Psychological Corporation.1999 Reference Source

[ref-27] Valentine GH: The growth and development of six XYY children. In A. Robinson, HA. Lubs, & D. Bergsma (Eds.): *Sex chromosomal aneuploidy: prospective studies on children. Birth Defects Orig Artic Ser.*New York: Alan R Liss.1979;15(1):175–190. 444640

[ref-28] Wechsler D: Wechsler Abbreviated Scale of Intelligence. San Antonio: Psychological Corporation.1999 Reference Source

[ref-29] Wigby K D'Epagnier C Howell S: Expanding the phenotype of triple X syndrome: A comparison of prenatal versus postnatal diagnosis. *Am J Med Genet A.* 2016;170(11):2870–2881. 10.1002/ajmg.a.37688 27644018 PMC6501572

[ref-30] Wilson AC Bishop DVM: Sex chromosome trisomies are not associated with atypical lateralization for language. *Dev Med Child Neurol.* 2018;60(11):1132–1139. 10.1111/dmcn.13929 29888392 PMC6220794

[ref-31] Woodcock RW McGrew KS Mather N: Woodcock-Johnson III Tests of Cognitive Abilities. Rolling Meadows, IL: Riverside Publishing.2007.

