# Supplementary material for: Language phenotypes in children with sex chromosome trisomies
Source: Wellcome Open Res. 2019 Jan 28;3:143. Originally published 2018 Nov 8. [Version 2] doi: 10.12688/wellcomeopenres.14904.2 (PMC6376256; doi:10.12688/wellcomeopenres.14904.2)
Supplement: Supplementary file 1 [file wellcomeopenres-3-16388-s0004.tgz › 8cc1a99d-0bf9-4e44-9d2c-6aa8f053461e_Supplementary_material.docx]

# Supplementary Material

## Distribution of scores on individual psychometric measures.

Figure S1 shows beeswarm plots for individual test scores: Low Bias trisomy groups and Language Concern twin group. Note that on some tests there is a minimum scaled score that leads to a skewed distribution.


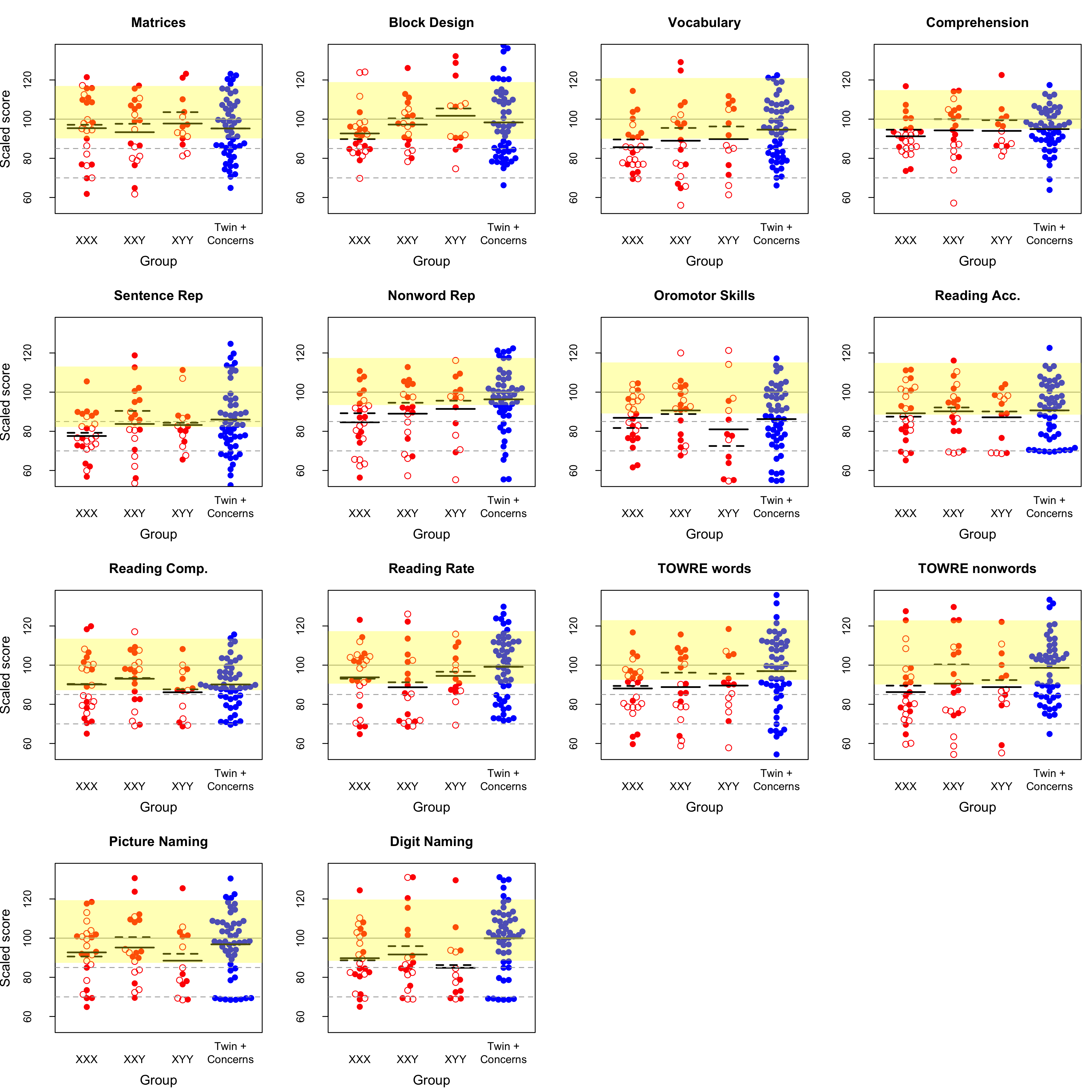


*Figure S1: Distributions of scores on the fourteen measures from psychometric tests for the Low Bias trisomy groups and the Language Concerns Comparison group. Open circles show cases in age range 6 to 11 yr. Solid line is mean for 6-11 yr olds, dotted line is mean for whole sample including those outside 6-11 yr age range. Yellow band is mean +/- 1 SD for No Concern comparison group.*

## CCC-2 subscale data

In the MANOVA for CCC-2, we used composites based on averaged subscales to minimise problems with non-normality of data. The data on the individual scales are summarised in Figure S2.


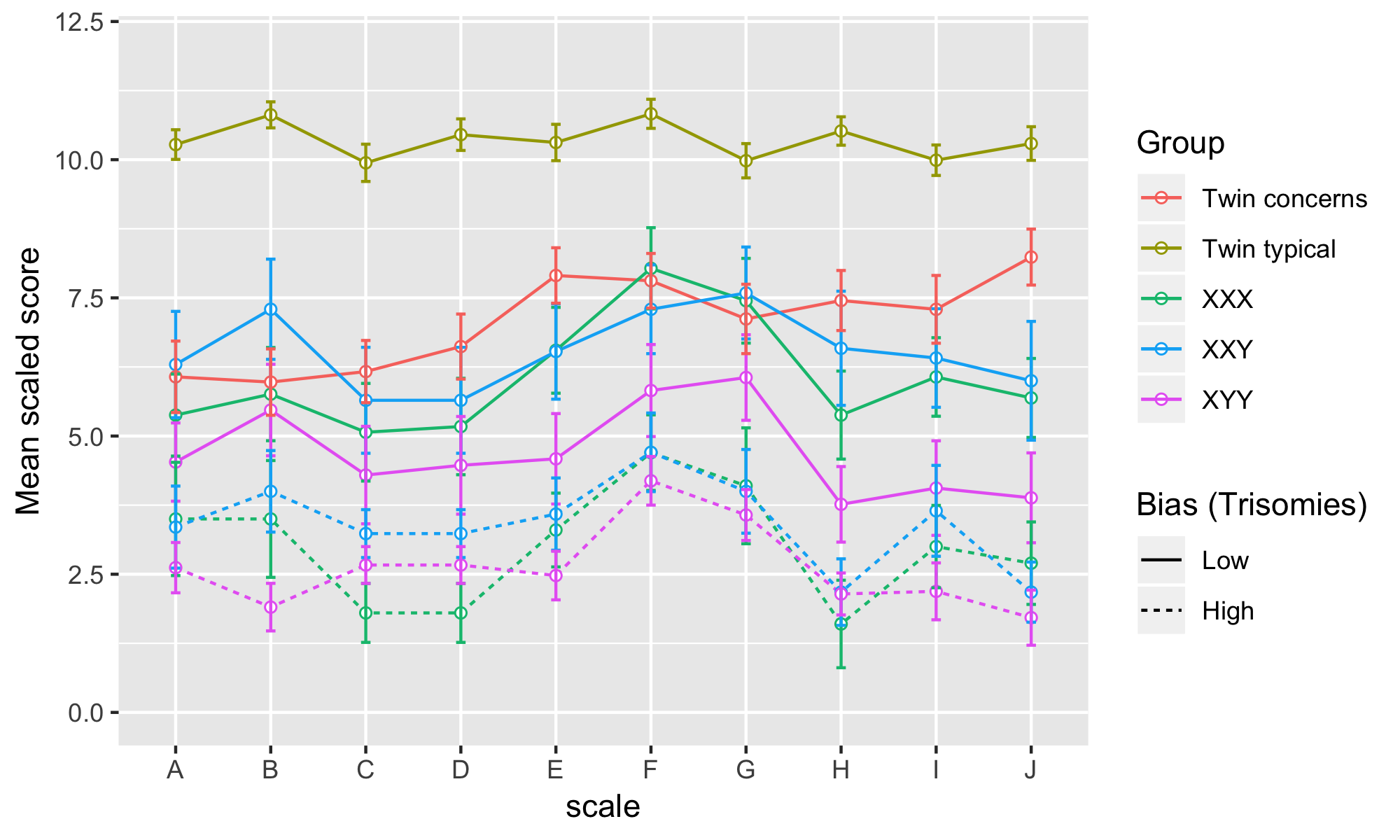


*Figure S2. Means for CCC-2 scales for trisomy groups subdivided by bias type (continuous lines = Low Bias, and dotted lines = High Bias) and for the two twin groups. Error bars show standard errors.*

## Prediction of CCC-2 checklist completion from child and parent variables.

Table S1 shows results of the logistic regression analysis predicting CCC-2 completion rate from parent and child variables.

*Table S1. Estimates of regression coefficients with CCC-2 completion rate as dependent variable, and parent and child variables as predictors.*

| Coefficient | Estimate | SE | z score | p-value |
| --- | --- | --- | --- | --- |
| Intercept | 1.289 | 0.347 | 3.713 | <.001 |
| Lang.severity | 0.096 | 0.033 | 2.937 | 0.003 |
| Mo_educ2 | 0.044 | 0.442 | 0.1 | 0.92 |
| Mo_educ3 | 0.803 | 0.52 | 1.544 | 0.123 |
| Mo_educ4 | 0.949 | 1.093 | 0.869 | 0.385 |
| Single parent | -0.173 | 0.461 | -0.376 | 0.707 |
| Trisomy/twin | 1.295 | 0.45 | 2.878 | 0.004 |

Figure S3 shows fitted points for CCC-2 response rate in trisomy and twin samples, in relation to language status of the child (score on language factor, where low score indicates impairment). Note that checklist completion was lowest for parents whose child had more severe language problems.


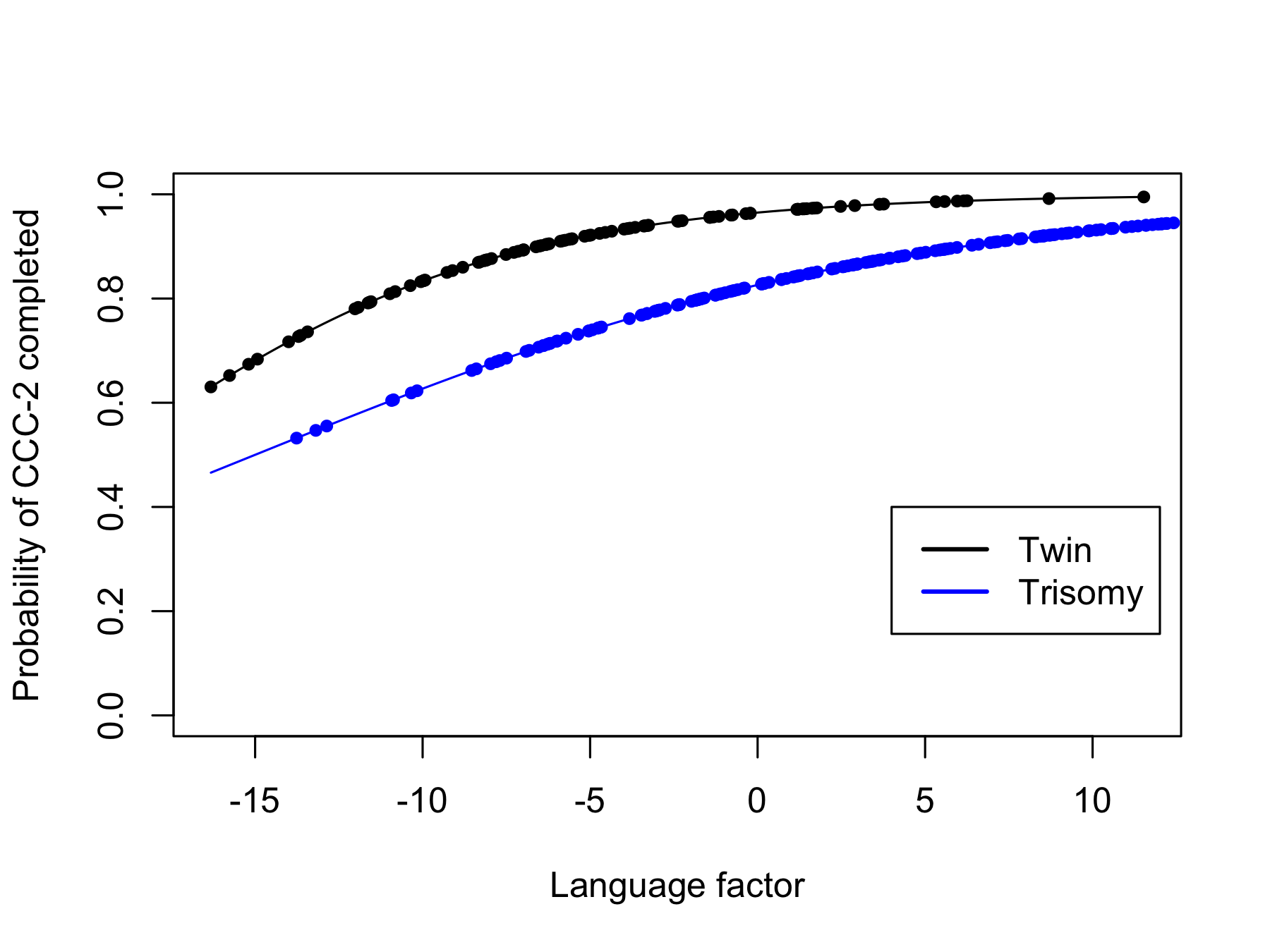


*Figure S3. Fitted regression lines for predicting CCC-2 completion from child status.*
