# Supplementary material for: Language phenotypes in children with sex chromosome trisomies
Source: Wellcome Open Res. 2019 Jan 28;3:143. Originally published 2018 Nov 8. [Version 2] doi: 10.12688/wellcomeopenres.14904.2 (PMC6376256; doi:10.12688/wellcomeopenres.14904.2)
Supplement: Peer review history

# Peer review: Reviewer response for version 2

**DOI:** 10.21956/wellcomeopenres.16388.r34705

**Published:** 2019-03-18

**Authors:** Nancy Raitano Lee

**Affiliations:** 1 Department of Psychology, Drexel University, Philadelphia, PA, USA

I was delighted to review “Language phenotypes in children with sex chromosome trisomies” for Wellcome Open Research. Bishop and colleagues describe a carefully designed study of the language outcomes of children with sex chromosome trisomies (SCTs; 47,XXX, 47,XXY, 47,XYY) whose genetic diagnoses were ascertained either through (a) prenatal screening or other medical investigations (‘low bias group’) or (b) during the course of testing to identify the etiology of neurodevelopmental problems (‘high bias group’). The study sought to answer three questions. First, does the profile and severity of language difficulties experienced by children with SCTs differ across the trisomy variants? Second, does the language phenotype associated with SCTs differ from that observed in children without known chromosomal abnormalities whose parents expressed concerns about their language? Third, to what extent does ascertainment bias affect language and cognitive skills in youth with SCTs?

 Findings can briefly be summarized as follows. First, the authors reported no statistically significant differences between SCT groups on language and cognitive testing. Second, the authors report similar language impairment severity and profiles for children with SCTs as compared to children from an existing twin sample whose parents expressed concerns about language development. Lastly, the authors noted that ascertainment bias appears to relate to the severity of language difficulties experienced by children with SCTs – that is, children from the high bias group showed greater language impairments than children from the low bias group.

 This research has several noteworthy strengths. I will highlight three here. First, the authors took great care to classify participants based on how the child’s SCT was ascertained. Because SCTs are often not associated with frank dysmorphology, they can go undiagnosed ^1^. Thus, a concern for studies examining language and learning problems in youth with SCTs is that they are only describing a subset of individuals whose diagnoses were discovered due to learning challenges. Second, the authors measured and covaried family background characteristics (mother’s education and multiple deprivation index) when evaluating study questions. As research suggests that family characteristics ^2^ and early life stress ^3^ relate to outcomes in children with SCTs, the inclusion of maternal education and adversity was an important methodological control implemented by these authors. Third, the authors included a comparison group of children with idiopathic developmental language concerns. In order to begin to understand whether children with SCTs and language impairments may benefit from similar interventions to those currently implemented for children with idiopathic language difficulties, direct comparisons of these groups are necessary.

 My review of this methodologically rigorous research study also raised several questions that I hoped the authors could address in this or future research. I will briefly mention two of these. The first involves the question of ascertainment bias. While I wholeheartedly agree with the authors that the manner in which a child’s SCT diagnosis is ascertained has the potential to bias study findings, I wonder if it is worth discussing the different types of bias involved with the low and high bias groups in greater detail. While the authors were careful to note that the low bias group was not free of bias, I had numerous questions about this group and whether they represent a less biased or rather differently biased group? In particular, I had questions about the subset of individuals in this group whose SCT was detected during prenatal testing. There appears to be multiple factors associated with who is offered and completes prenatal testing. The authors note that one factor is advanced maternal age. Based on this, I had the following questions. What were the ages of the mothers in the two groups? Were the mothers of the children in the low bias group older? Recent research suggests that older mothers experience greater (or perhaps more persistent) happiness after birth than younger mothers ^4^. As there is research linking maternal mental health to child cognitive/linguistic development ^5^, I wondered if the participants in the low bias group may have experienced advantages that could have influenced their language development favorably (above and beyond those that were controlled for statistically in the study’s analyses). From my brief review of the literature, another factor that appears to relate to prenatal testing rates in the United Kingdom is ethnicity ^6^. Based on this research, I hoped that the authors could address whether there was an even distribution of children from multilingual families across these groups? (I assume that this was the case, but I could not find specific mention of this in the Method). Lastly, I wondered about differences in the receipt of speech/language therapy services between the groups. Do the authors have information about any preventative or early speech-language therapy services that may have been sought for the participants in the low bias group who were diagnosed prenatally? I recognize that it is quite difficult to quantify the type and dose of treatment a child may have received retrospectively. However, I do wonder if some discussion of service provision for the different groups would be helpful to include.

 My second set of questions relates to whether the authors have or will examine relations among different aspects of cognitive functioning (e.g., nonverbal IQ, verbal short-term memory) and language outcomes in the children with SCTs. In particular, I had questions about whether there were any differences in the strength of the relationships between different cognitive abilities and language skills in the youth with SCTs as compared to those with idiopathic language concerns. In recent work from my lab looking at autism spectrum disorder (ASD) symptoms in youth with idiopathic ASD and those with ASD within the context of Down syndrome (Godfrey et al., *in press* ^7^), there was a suggestion (that we were not powered to evaluate statistically due to small sample sizes) of a differential relationship between verbal mental age and social communication impairments for children with ASD that was idiopathic in nature or occurred within the context of Down syndrome. Specifically, there appeared to be a tighter coupling between verbal mental age and social communication in children with Down syndrome and ASD than in the idiopathic ASD group. Have Bishop and colleagues examined relations between nonverbal IQ, verbal short-term memory, and language functioning in this sample? I raise this question, as (a) there may be differences in the cognitive underpinnings or correlates of language challenges in those with SCTs as compared to those with idiopathic language problems, and (b) differences in these relationships may be of use to consider when planning and implementing treatments for children with SCTs.

 Having noted my questions for the authors, I’d like to close by thanking Dr. Bishop and colleagues for inviting me to review this work. I enjoyed reviewing this manuscript and I look forward to the papers to come on this unique sample of children with SCTs.

I have read this submission. I believe that I have an appropriate level of expertise to confirm that it is of an acceptable scientific standard.

## References

[1] BoydPALoaneMGarneEKhoshnoodBDolkHEUROCAT working group: Sex chromosome trisomies in Europe: prevalence, prenatal detection and outcome of pregnancy. *Eur J Hum Genet*.2011;19(2) : 10.1038/ejhg.2010.148 231-4 10.1038/ejhg.2010.148 20736977PMC3025783

[2] BenderBGHarmonRJLindenMGRobinsonA: Psychosocial adaptation of 39 adolescents with sex chromosome abnormalities. *Pediatrics*.1995;96(2 Pt 1) :302-8 7630689

[3] van RijnSBarneveldPDescheemaekerMJGiltayJSwaabH: The effect of early life stress on the cognitive phenotype of children with an extra X chromosome (47,XXY/47,XXX). *Child Neuropsychol*.2018;24(2) : 10.1080/09297049.2016.1252320 277-286 10.1080/09297049.2016.1252320 27892807

[4] MyrskyläMBarclayKGoisisA: Advantages of later motherhood. *Gynakologe*.2017;50(10) : 10.1007/s00129-017-4124-1 767-772 10.1007/s00129-017-4124-1 29070913PMC5633623

[5] AhunMNCôtéSM: Maternal depressive symptoms and early childhood cognitive development: a review of putative environmental mediators. *Arch Womens Ment Health*.2019;22(1) : 10.1007/s00737-018-0870-x 15-24 10.1007/s00737-018-0870-x 29876681

[6] RoweREGarciaJDavidsonLL: Social and ethnic inequalities in the offer and uptake of prenatal screening and diagnosis in the UK: a systematic review. *Public Health*.2004;118(3) : 10.1016/j.puhe.2003.08.004 177-89 10.1016/j.puhe.2003.08.004 15003407

[7] GodfreyMHepburnSFidlerDTaperaTZhangFRosenbergCLeeNR: Autism spectrum disorder (ASD) symptom profiles of children with comorbid Down syndrome (DS) and ASD: A comparison with children with DS only and ASD only. *Research in Developmental Disabilities*.(in press);10.1016/j.ridd.2019.03.00330959431

# Peer review: Reviewer response for version 2

**DOI:** 10.21956/wellcomeopenres.16388.r34707

**Published:** 2019-02-14

**Authors:** Laura Zampini

**Affiliations:** 1 Department of Psychology, University of Milano-Bicocca, Milan, Italy

It is a great pleasure for me to read and comment on this interesting and important paper. I recently started studying language development in Italian children with sex chromosome trisomies and the study of Bishop *et al.* is an excellent model for investigating this topic.

 In the present study, Bishop *et al. *analysed language development in children with sex chromosome trisomies comparing their language profile with that of children with language disorders of unknown cause. After excluding children with autism, intellectual disability or hearing problems, the authors found that children with sex chromosome trisomies identified via prenatal screening had an increased risk of language problems, but severe language disorders were not common in this specific group.

 The study has many strengths, among which the wide number of participants with sex chromosome trisomies (N = 142). The methods are appropriate. One of the merits is the division of children into two groups: those identified by prenatal screening (Low bias) and those diagnosed during postnatal testing for neurodevelopmental problems (High bias). Controlling for children’s socio-economic background is also of great value.

 Moreover, the study has improved after the first revision, in particular with the inclusion of information about the excluded cases (i.e., children with autism and intellectual disability) in the beeswarm plots.

 Data analyses are detailed, and the results are clearly reported.

 My only concern is about the conclusions drawn by the study. The argumentation of the results, in the abstract and the discussion, is in my opinion too optimistic for the families of these children. It is true that around one-third of the children identified by prenatal screening does not show language disorders. However, about 17% of children in the Low bias group have been excluded due to a developmental disorder (i.e., autism, intellectual disability, hearing impairment). Furthermore, a high percentage of children with a prenatal diagnosis scored more than 1 SD below normative data in at least one area of language development (only 10.7% of girls with XXX and 27.8% of children with XXY did not show any significant problem).

 Moreover, I wonder if this optimistic conclusion could also be related to the participant’s age.

 In our recent study ^1^, although with a very limited number of participants (i.e., 15 24-month-old children), we found that 60% of children with sex chromosome trisomies (75% of children with XXY and 43% of children with XXX) were at risk for language impairments (i.e., they had a vocabulary size lower than 50 words at 24 months). The current follow-up on these children (data collection in progress) is showing that at 4 years almost all the late-talking children with sex chromosome trisomies meet the criteria for a diagnosis of language impairment.

 Is it possible that some of the participants in the present study (in particular the oldest ones) have partially compensated for their linguistic problems?

 One important missing information is the number of children with sex chromosome trisomies in both groups (Low and High bias) that had (or have) speech and language therapy. How many children undergo rehabilitation treatments? For how long? These data should be reported since it is possible that the involvement in speech and language therapy could influence the children’s performance on standardised tests.

I have read this submission. I believe that I have an appropriate level of expertise to confirm that it is of an acceptable scientific standard.

## References

[1] ZampiniLDraghiLSilibelloGDall'AraFRigamontiCSuttoraCZanchiPSalerniNLalattaFVizzielloP: Vocal and gestural productions of 24-month-old children with sex chromosome trisomies. *Int J Lang Commun Disord*.53(1) : 10.1111/1460-6984.12334 171-181 10.1111/1460-6984.12334 28726283

# Peer review: Reviewer response for version 2

**DOI:** 10.21956/wellcomeopenres.16388.r34701

**Published:** 2019-01-29

**Authors:** Kate Baker

**Affiliations:** 1 MRC Cognition and Brain Sciences Unit, and Department of Medical Genetics, University of Cambridge, Cambridge, UK

No new comments. I concur entirely with the balanced view detailed within the response to my initial comments.  I am sincerely grateful for the opportunities to learn via this review process.

I have read this submission. I believe that I have an appropriate level of expertise to confirm that it is of an acceptable scientific standard.

# Peer review: Reviewer response for version 1

**DOI:** 10.21956/wellcomeopenres.16247.r34202

**Published:** 2018-11-27

**Authors:** Kate Baker

**Affiliations:** 1 MRC Cognition and Brain Sciences Unit, and Department of Medical Genetics, University of Cambridge, Cambridge, UK

Investigating the associations between rare genomic variants and developmental cognitive impairments is of ever-growing importance.  The number of genomic tests being carried out is increasing rapidly, at any point from early prenatal to adulthood.  The proportion of genomic tests reported as abnormal is also increasing, as the catalogue of disorder-associated genes grows week by week and the analysis of chromosomal variants and sequence variants improves.

 After a genetic diagnosis, three questions usually arise: To what extent does this diagnosis explain the individual’s past and present problems (taking into account other risk factors they may also be exposed to)?  To what extent does this diagnosis predict the types and severities of future problems for this individual? To what extent should this genetic diagnosis be taken into account when planning interventions for this individual?  In other words, how much difference can or should knowledge of genetic cause really make? 

 At present we lack the evidence base to answer these questions, to make genetic testing count for patients and their families, and to insure that genetic diagnosis is helpful not harmful. The limited literature is rife with claims of specific associations between rare genotypes and developmental profiles, claims which lack robustness in multiple respects – theoretically unsound, underpowered studies, highly biased samples, lack of informative comparison groups, inadequate phenotyping measures and exploratory statistical analysis.  The field has not yet embraced open science.

 This paper by Bishop *et al* is of high importance, for our understanding of sex chromosome trisomies (SCTs) in particular, but also for genomic disorders more broadly.  The study raises the bar for this research field, via transparent study design, reporting and open data.  These results should inform post-diagnostic genetic counselling for SCTs, and influence the future research agenda for these groups and other genomic disorders.

 The paper tackles three major theoretical and clinical problems – specificity of associations between genomic disorders (three different SCTs) and developmental outcomes (language impairments), specificity of associations between genomic and non-genomic risk factors (SCT vs twins with language concerns), and the influence of ascertainment bias on SCT data. To summarise the findings, the authors confirm that SCT is a risk factor for developmental language impairment but find no evidence for specificity of outcomes between genomic disorders, no evidence for specificity of outcomes between genomic and non-genomic disorders. They find further evidence that ascertainment bias predicts severity of impairments within SCTs.

 However, there are some important limitations in drawing these conclusions, which I feel could be discussed more directly and fully in order to maximise the utility and impact of this paper:

 1.   Are the SCT groups reported in the paper truly representative?

- The low bias group was mainly detected prenatally.  Indications for prenatal testing include abnormalities on fetal ultrasound and abnormal first trimester screening (to a greater extent than advanced maternal age), which are early indicators of developmental risk.
- For both low and high bias groups, only children aware of their genetic diagnosis could be invited to the study (an inclusion criterion likely to be predictive of developmental risk).  Families volunteering for this research were likely to be motivated by concerns about their children’s development, biasing both groups toward impairment.
- I understand and appreciate the inclusion / exclusion criteria applied to compare the SCT groups to the control samples.  However a very high proportion of the SCT groups were excluded due to intellectual disability and autism, which are deeply connected to the outcomes under investigation.  For the high bias group in particular, these criteria led to more than one third of the sample being excluded, and a further third can unusable data.  The full spectrum of impairments in the SCT group is therefore heavily under-estimated.  Would it be possible for the authors to provide more information within the paper about the excluded SCT participants, and discuss whether incorporating these individuals would be likely to alter their main findings?

2.  Is the reported data sufficient for validity and interpretation of results?

- The biggest limitation here is the high proportion of missing data for both neuropsychological and questionnaire assessments, meaning that only mildly impaired individuals are included in statistical analysis.  This is openly reported, but it is somewhat difficult to appraise the quantities of missing data across analyses and I wonder if the authors could summarise this more clearly.  For example, Table 2 provides descriptive demographic data for all participants (with suggestion that the high bias SCT groups may be associated with lower maternal education, lower neighbourhood advantage and higher family history scores).  However if this data was displayed in addition for the subset of participants (defined by age and data availability) included in statistical comparison of (a) neuropsychological assessments and (b) CCC-2, this might indicate whether there were potential non-genomic biases in the available datasets and results. 
- Connected, the high rates of missing data emphasise the need to improve methods that can be applied to children with intellectual impairments.  Put more simply, it is difficult to draw firm conclusions about the nature and severity of language impairments if a large proportion of individuals with the very communication deficits of interest cannot be investigated.  Could the authors discuss whether this limitation challenges their conclusions?  And could they make some suggestions for new approaches which could tackle this issue head-on?

3.  Are there other potential sources of false negative results to consider?

- Can the authors comment on the power that would be needed to detect significant differences within the analyses that they present?  I appreciate the immense work that the team dedicated to this study and the impressive total sample size of rare disorders that they amassed over a five year period. The results should certainly be published.  However, should interpretation of results dependent on a small subset of the total population be cautious?
- Inspecting the results in detail I note in Table 4, final row lists “Bias x Trisomy” but no results are given.  This omission speaks to the question of whether there may be informative interactions yielding hypotheses for future studies, which could be explored with necessary caution.  For example, Figure 4 suggests that bias within the SCT sample may have limited influence on core language skills or literacy skills, but impact on nonverbal ability and verbal production / memory.  Furthermore, the impact of bias on these domains appears most marked for participants with XXX.  On the other hand, participants with XYY demonstrate the most severe pragmatic and autistic features on CCC-2, in both the low and high bias groups.  Would it therefore be wrong to conclude at this point that there are definitely no specific associations between SCTs and risk profiles, which may not be clinically predictive in view of high within-group variability but could nevertheless suggest differences in underlying mechanisms?

4. Is the concept of “typical” outcomes and profiles valid or useful?

- The abstract refers to typical outcomes in the Background and Conclusions.  I wonder whether this concept is supported by the data, and whether either the abstract be reworded or the discussion extended to tackle this idea head-on.

I have read this submission. I believe that I have an appropriate level of expertise to confirm that it is of an acceptable scientific standard.

# Response

**Published:** 2019-01-05

**Authors:** Dorothy Bishop

**Affiliations:** University of Oxford, UK

We thank the reviewer for her helpful comments, which are useful not just for this paper, but also for our forthcoming companion paper that focuses more on ASD and psychiatric aspects.  In the course of addressing the comments, we discovered some errors in the reported numbers in different subsets of the sample, which had arisen because of confusion about the coding of variable names. This was the responsibility of DVMB, who is embarrassed at the errors, but glad of the opportunity to correct them – and grateful to the reviewer for noting inconsistencies that led to their discovery.

**Sampling bias**

The first question raised by the reviewer is whether  the SCT groups reported in the paper are truly representative. She notes that children diagnosed prenatally will have had indications for prenatal testing, that volunteer families may be atypical, and that children with ASD and intellectual disability were excluded.

We have now added more information about the Low Bias group, as follows:

*This latter group included 49 families who underwent amniocentesis: all but 4 parents provided a reason: 30 because of maternal age, 11 because nuchal screening or ultrasound scan indicated risk of a chromosome disorder, and 4 because of a family history of a chromosome disorder. A further 23 children had genetic testing for a range of medical conditions, including growth problems, failure to thrive, and failure to go through puberty (in the case of boys with XXY). Note that in referring to these as a ‘Low Bias’ group, we do not imply they are a representative sample of children with an extra sex chromosome: we make the more limited claim that the trisomy was not discovered as a result of the child manifesting language or behaviour difficulties. All genetic testing was done via the National Health Service, so the sample was not limited by parents’ ability to pay for a test.*

The issue of volunteer bias is also very pertinent. This can cut both ways: as the reviewer notes, some parents may volunteer because they hope to obtain more information about their child's difficulties. On the other hand, some seemed keen to take part to emphasise how well their child was functioning, especially if they had been given an unrealistically poor prognosis at the time of diagnosis. The child's participation is also an issue. As we noted in our discussion, we previously found that parents are more likely to tell their child about the trisomy if the child is experiencing difficulties (Gratton et al, 2016), in which case the child can feel a sense of relief at having an explanation for why they are different from others. On the other hand, there is likely to be an opposing potential bias, which is that children who are anxious about social or communicative difficulties are less likely to assent to take part, even if parents want them to.

Could these problems be overcome? Potentially, one could conduct another study that involved neonatal screening, to complement the studies done in the 1960s, but even if ethics committees approved such a study, there would still be issues about bias among those who consented to take part. An interesting example comes from a study was published by Tuke et al (2018), reporting on characteristics of women from the Biobank study who were discovered to have a sex chromosome trisomy. They were reported to be taller than average, to have earlier menopause, and to perform more poorly on a test of intellectual ability than other women of normal karyotype. Correspondence with the authors confirmed that none of these women were aware of the trisomy (or at least, none of them reported it on a medical history screen). At first glance, this looks like a perfect way of identifying a truly representative sample. However, there is volunteer bias in Biobank: only 5% of those invited to take part agreed to do so, and the response rate was more than twice as high for those from affluent areas than from areas of social disadvantage (Fry et al, 2018).  Furthermore, Tuke et al (2018) identified 110 women with 47,XXX among 244,848 women in Biobank, which is less than half the number expected on the basis of estimated prevalence of around 1 in 1000 (Nielsen & Wohlert, 1991).  We know that adults with trisomy X tend to have relatively unskilled occupations and it seems likely that many may prefer not to participate in the Biobank study. These points are made not to criticise studies based on Biobank, but merely to note that recruitment of a truly representative sample is never possible, because those who consent will have certain characteristics, and one cannot compel people to take part in research. Given that is the case, the key issue is to be open about possible sources of bias and their likely impact.

It is also important to consider what question is being addressed, and how the information will be used. This is particularly important when families may be offered a termination of pregnancy if the trisomy is discovered prenatally, as we know that their decision can be heavily influenced by what they are told about outcomes (Jeon et al, 2012). For sex chromosome trisomies, there are enduring concerns similar to those expressed by Linden et al (2002), who note that parents seeking information on the internet may be confronted by information that either exaggerates the problems, or is factually wrong. Our interest is in language disorders, but we feel it is very important to draw attention to the fact that many children from the Low Bias sample had relatively mild language problems, with some scoring within normal limits. We noted the potential for bias toward impairment in our sample for this reason, but even taking that into account, there were many children who did well.

**Exclusions**

In terms of the point about exclusions, it is important to recognise that our main focus is on the Low Bias group, where exclusions applied to 16% of cases (corrected data).  The exclusion rate was high in the High Bias group,  but our main purpose in including that group was to estimate the bias that was introduced by including such cases. As we note in the paper, the differences between the two groups are substantial, even with ASD/ID cases excluded: clearly they would be even greater if we had included them. We make that point, but do not feel this compromises the main conclusion deriving from the Low Bias vs High Bias comparison – which is that it is very important to minimise bias as far as possible.

We accept the reviewer's point, that more information is needed about the excluded cases, rather than just referring to a future paper, and so in the current paper we have now included them in the beeswarm plots for the low-high bias comparison, distinctively marked, so readers can get an impression of the impact of the exclusion. We have not included them in the MANOVA, as the principal focus is the comparison of the SCT and twin samples in terms of language profile, and ASD/ID cases were not included in the twin sample.

One other note: since submitting this paper, we have been working on a manuscript documenting ASD and social anxiety in these children, and for consistency with that paper, there has been a change to the coding of ASD in the current paper. A subset of children were assessed using the Development and Wellbeing Assessment, and we had previously used one rater's diagnosis for ASD. We now have consensus data, which sometimes gave slightly different results, and this is what is used in the latest version of the paper.

The final paragraph, with clinical recommendations, has been modified to take into account these points about ascertainment bias.

**Missing data**

The statement that 'only mildly impaired individuals are included in statistical analysis' represents a misunderstanding. It is evident from the beeswarm plots, that show the whole range of scores for all individuals, that the sample included children who scored very poorly – see, for instance, Figure 4, which shows many children who scored more than 2 SD below the mean.  This was, however, confusingly reported, and in checking all the tables and figures, we realised there were some errors in the reporting. We thank the reviewer for picking this up, making it possible for us to make some corrections, as follows:

1. Figures 1 and 2 were intended to give a complete picture of the numbers of children at each point in the analysis for each sample, but a gremlin got into the script, which meant that some numbers were computed from the wrong subset of data. This has now been corrected.

2. Table 2 should have just shown the demographic data for the children included in the language test analysis, whereas in fact it showed data for the whole sample, including cases who had been excluded. This has now been corrected.

3. Because MANOVA required complete data for all children, any child with missing data on any of the 4 composites (PIQ, general language, verbal memory or literacy) was excluded from the analysis. Most missing data from the Low Risk group was simply because the children were too young for test norms. Indeed, our original goal had been to include only those aged 6 years and over, but given that we had some 5-year-olds volunteered for the study, and it would be feasible to collect a subset of data with them, including the CCC-2, we decided to reduce the lower age limit to 5 years towards the end of the study. So it would be wrong to conclude that these children could not attempt the tests. A second point is that for the majority of other children excluded from MANOVA, there was missing data on just one composite, and this was usually the Literacy composite. To give a slightly larger sample on MANOVA, we decided to exclude literacy from that analysis; we now report a separate ANOVA for Literacy. In addition we note the numbers of cases with missing data, distinguishing between those out of age range for norms, and those who did not complete the tests.

We thank the reviewer for drawing our attention to this, because it is clear that by focussing attention only on children who completed the whole battery, we gave the misleading impression that many children could not be tested, which was not the case. In the High Bias group there were some children who were more challenging to assess and who were unwilling or unable to attempt certain tests. But as we stress throughout, the results from the High Bias group need to be treated with extreme caution: our main reason for including them is to show how ascertainment bias can distort results.

So in sum, the beeswarm plots have now been amended to include children who had partial data.  We had previously included only those children who were in the MANOVA so that the beeswarm data would match the MANOVA data, and show the relevant means. We are still able to show that information, although it does make the plot a bit more complicated.

**Power**

The reviewer raises an important point: all studies in this area have been beset by small sample sizes, and it is likely that this is why findings of karyotype-specific effects have been inconsistent. We have now added this comment in our discussion:

*Note, however, that detection of differences in variance between groups would require substantial sample sizes. Even for detection of mean group differences, our sample size was too small to detect any but large effects of karyotype: for comparisons of XXX+XXY vs XYY we had 80% power to detect an effect size on psychometric composites of .83, and on CCC-2 an effect size of .97. We cannot therefore conclude from the current study that there are no sex-chromosome-specific influences, only that if they do exist, they are not large, and are superimposed on substantial variation from other sources. Because of the difficulty of recruiting large samples, and the difficulty of controlling sampling biases, we will need to combine samples across different research groups to get a clear answer to the question of whether an additional X or Y has consistently different effects.*

**Table 4**

Apologies: There was an error in Table 4 with a misleading row added. We did not look at the interaction between trisomy and bias group as this would not address the questions of interest. (And also the power would be abysmal even if there was interest in such an interaction). Our data suggested there could be differences between trisomies in terms of who gets into the High Bias group, but that was a post hoc observation, based on finding that the numbers depart from expectation, with relatively few XXX girls in the High Bias group. The chi square analysis we did on the Ns in the 2 x 3 table draw attention to this imbalance, which we think is the best way to flag this up.

**Is the concept of “typical” outcomes and profiles valid or useful?**

This is a valid comment: our goal was really to try and distinguish what were the most common profiles seen in sex chromosome trisomies, though given the enormous heterogeneity it is perhaps unhelpful to talk of anything being 'typical'. We say a bit more about implications for genetic counselling: perhaps the message for parents is exactly this – there is no 'typical'. Nevertheless, they need to be given some idea of the range of possibilities. We refer back to the 2002 paper of Linden et al on genetic counselling; we feel our paper supports their recommendations regarding a condition that is very challenging to advise on.

References

Fry, A., Littlejohns, T. J., Sudlow, C., Doherty, N., Adamska, L., Sprosen, T., . . . Allen, N. E. (2017). Comparison of sociodemographic and health-related characteristics of UK Biobank participants with those of the general population. *American Journal of Epidemiology, 186*(9), 1026-1034. doi:https://doi.org/10.1093/aje/kwx246

Gratton, N. C., Myring, J., Middlemiss, P., Shears, D., Wellesley, D., Wynn, S., . . . Scerif, G. (2016). Children with sex chromosome trisomies: parental disclosure of genetic status. *European Journal of Human Genetics, 24*(5), 638-644. doi:10.1038/ejhg.2015.168

Jeon, K. C., Chen, L.-S., & Goodson, P. (2012). Decision to abort after a prenatal diagnosis of sex chromosome abnormality: a systematic review of the literature. *Genetics in Medicine, 14*, 27-38.

Linden, M. G., Bender, B. G., & Robinson, A. (2002). Genetic counseling for sex chromosome abnormalities. *American Journal of Medical genetics, 110*(1), 3-10. doi:10.1002/ajmg.10391

Nielsen, J., & Wohlert, M. (1991). Chromosome abnormalities found among 34910 newborn children: results from a 13-year incidence study in Århus, Denmark. *Human Genetics, 87*, 81-83.

Tuke, M. A., Ruth, K. S., Wood, A. R., Beaumont, R. N., Tyrrell, J., Jones, S. E., . . . Murray, A. (2018). Mosaic Turner syndrome shows reduced penetrance in an adult population study. *Genetics in Medicine*. doi:https://doi.org/10.1038/s41436-018-0271-6
